# PINK1 Is Necessary for Long Term Survival and Mitochondrial Function in Human Dopaminergic Neurons

**DOI:** 10.1371/journal.pone.0002455

**Published:** 2008-06-18

**Authors:** Alison Wood-Kaczmar, Sonia Gandhi, Zhi Yao, Andrey S. Y. Abramov, Erik A. Miljan, Gregory Keen, Lee Stanyer, Iain Hargreaves, Kristina Klupsch, Emma Deas, Julian Downward, Louise Mansfield, Parmjit Jat, Joanne Taylor, Simon Heales, Michael R. Duchen, David Latchman, Sarah J. Tabrizi, Nicholas W. Wood

**Affiliations:** 1 Department of Molecular Neuroscience, Institute of Neurology, London, United Kingdom; 2 Medical Molecular Biology Unit, Institute of Child Health, London, United Kingdom; 3 Department of Physiology, University College London, London, United Kingdom; 4 ReNeuron Ltd, Guildford, United Kingdom; 5 Eisai London Research Laboratories Ltd, London, United Kingdom; 6 Neurometabolic Unit, National Hospital for Neurology and Neurosurgery, London, United Kingdom; 7 Cancer Research United Kingdom, London, United Kingdom; 8 Department of Neurodegenerative Disease, Institute of Neurology, London, United Kingdom; 9 Birkbeck, University of London, London, United Kingdom; University of Auckland, New Zealand

## Abstract

Parkinson's disease (PD) is a common age-related neurodegenerative disease and it is critical to develop models which recapitulate the pathogenic process including the effect of the ageing process. Although the pathogenesis of sporadic PD is unknown, the identification of the mendelian genetic factor PINK1 has provided new mechanistic insights. In order to investigate the role of PINK1 in Parkinson's disease, we studied PINK1 loss of function in human and primary mouse neurons. Using RNAi, we created stable PINK1 knockdown in human dopaminergic neurons differentiated from foetal ventral mesencephalon stem cells, as well as in an immortalised human neuroblastoma cell line. We sought to validate our findings in primary neurons derived from a transgenic PINK1 knockout mouse. For the first time we demonstrate an age dependent neurodegenerative phenotype in human and mouse neurons. PINK1 deficiency leads to reduced long-term viability in human neurons, which die via the mitochondrial apoptosis pathway. Human neurons lacking PINK1 demonstrate features of marked oxidative stress with widespread mitochondrial dysfunction and abnormal mitochondrial morphology. We report that PINK1 plays a neuroprotective role in the mitochondria of mammalian neurons, especially against stress such as staurosporine. In addition we provide evidence that cellular compensatory mechanisms such as mitochondrial biogenesis and upregulation of lysosomal degradation pathways occur in PINK1 deficiency. The phenotypic effects of PINK1 loss-of-function described here in mammalian neurons provides mechanistic insight into the age-related degeneration of nigral dopaminergic neurons seen in PD.

## Introduction

Parkinson's disease (PD) is the most common neurodegenerative motor disorder in the Western world. The disease is characterised clinically by resting tremor, rigidity and slowness of movement (bradykinesia) with symptoms being partially alleviated by administration of exogenous dopamine (L-dopa). Upon neuropathological examination, the brains of patients with PD show marked loss of pigmented dopaminergic (DAergic) neurons of the substantia nigra pars compacta (SNpc), and other brain regions. In addition, surviving neurons frequently contain Lewy bodies (LBs) which are intracytoplasmic proteinaceous inclusions, predominantly composed of aggregated α-synuclein. PD is a progressive, incurable and age-related disease, affecting ∼1.8% individuals by the age of 65 years [1]. The majority (>85%) of PD cases are sporadic, and the underlying molecular causes unknown. Insight into the mechanisms of PD pathogenesis has come from the identification of mutations in genes associated principally with familial forms of PD. Inherited forms of PD have been linked to mutations in six different genes with seemingly diverse functions. These encode the synaptic protein α-synuclein [2]; an E3 ubiquitin ligase, parkin [3]; a putative antioxidant chaperone, DJ-1 [4], a mitochondrial kinase, (PTEN)-induced kinase 1 (PINK1) [5], a mitochondrial serine protease, OMI/HTRA2 [6], and leucine-rich repeat kinase 2 (LRRK2) [7]. Discovery of these genes have strongly implicated certain cellular processes in the etiology of both inherited and sporadic disease; namely protein aggregation and impairment of the ubiquitin proteasome system, mitochondrial dysfunction, oxidative stress and protein phosphorylation [8].

The role of mitochondrial dysfunction in PD has been suggested since the original discovery that the complex I inhibitor 1-methyl-4-phenyl-1,2,3,6-tetrahydropyridine (MPTP) caused the development of PD in recreational drug users [9]. Other complex I inhibitors including rotenone and paraquat have similarly been found to cause PD–like symptoms in rodent models [10]. Inhibition of the mitochondrial respiratory chain is known to increase the generation of free radicals leading to cellular oxidative stress within cells [11]. Concordantly, evidence of impaired complex I activity has been reported in post-mortem PD brain tissue with an increase in markers of oxidative stress [12]. However the most convincing evidence to date has been the characterisation of genes mutated in familial PD with putative functional roles within mitochondria. Both PINK1 and Omi/HtrA2 have predicted mitochondrial targeting sequences and appear to exhibit protective functions within this organelle [5], [6], [13]. Parkin, DJ-1, α-synuclein and LRRK2 have also been shown to associate with the mitochondrion or impact upon its function, but the mechanisms involved remain unclear [14]–[17].

PINK1 is a highly conserved 581 amino acid protein with a catalytic serine/threonine kinase domain with close sequence homology to CAMK1 [5]. Several studies have demonstrated that recombinant PINK1 can undergo autophosphorylation as well as phosphorylate generic substrates *in vitro* [18]–[20]. It has a predicted N-terminal mitochondrial targeting motif and a significant proportion has been localised to both the inner and outer mitochondrial membranes [5], [19], [21]. Full length PINK1 pre-protein (∼63 kDa) can also be cleaved to a ‘mature’ form (54 kDa) by an unknown protease [21], [22]. PINK1 mRNA is ubiquitously expressed in human tissues, with highest expression in heart, muscle and testes [23]. It is uniformly expressed in mammalian brain, with highest expression levels found within the cell bodies of neurons and glia [21], [24].

Homozygous and compound heterozygous mutations in the PINK1 gene locus are known to cause PARK6 familial Parkinsonism, which is indistinguishable from idiopathic PD apart from an earlier age of onset [5]. To date there are no neuropathological data from any individual affected with a homozygous mutation in the PINK1 gene. However, brains of patients with PINK1 heterozygote mutations display the typical pathological hallmarks of idiopathic PD [21]. The prevalence of PINK1 mutations in autosomal recessive early onset PD range from 0-15%, depending on the patient series analysed [25]–[27]. This suggests that PINK1 is the second most common causative gene in early onset PD after parkin [25]. The vast majority of pathogenic mutations in PINK1 gene are located within the kinase domain and include nonsense, missense and deletion mutations which are predicted to either reduce or obliterate kinase activity [20]. Accordingly, reduced kinase activity has been demonstrated *in vitro* for the pathogenic mutations G309D, L437P, G386A and G409V [18], [20]. The most common mutation, a C1366T transition, reportedly triggers nonsense-mediated mRNA decay, resulting in a 80–90% reduction in transcript levels in tissues from homozygous patients [28]. Taken together, these findings suggest that PARK6 parkinsonism results from a loss-of-function of the PINK1 protein.

Valid animal models of PINK1 parkinsonism should recapitulate the motor symptoms seen in patients as well as the underlying nigrostriatal deficiencies and neuropathological findings. However, no motor impairments or other non-motor parkinsonian symptoms have been reported in PINK1 loss-of-function mouse models to date [29], [30]. In contrast, *Drosophila* models of PINK1 deficiency have given us closer insights into the molecular mechanisms involved [31]–[34]. Motor deficits include abnormal wing posture, rigidity, flight impairment and reduced climbing ability [31], [32], [34]. Moreover, some of these motor phenotypes were found to be progressive with age [32], [34] and showed partial but significant decreases (∼10%) in the numbers of dopaminergic neurons in certain key clusters within the fly brain [32]–[34]. Ultrastructurally, muscle fibres contained vacuolated, swollen and dysmorphic mitochondria, with disorganised christae which was also seen in surviving dopaminergic neurons [32]. Both ATP production and resistance to oxidative stressors was compromised in PINK1 deficient flies. Although fly models of PINK1 loss-of-function have recapitulated mitochondrial dysfunction and age-related neuronal death, the actual mechanisms remain unclear.

### Cellular pathogenesis of PINK1 loss of function in PD

The molecular mechanism by which PINK1 operates in the mitochondria is undefined, but the first *in vitro* cellular studies of PINK1 loss-of-function implicated a role for the kinase in maintenance of mitochondrial membrane potential and prevention of stress-induced apoptosis [5]. Over-expression of wild-type (but not pathogenic variants) in tumour cell lines was subsequently shown to prevent release of cytochrome c from mitochondria and caspase-3 activation during stress [5], [35], [36]. In contrast, depletion of PINK1 from cells using transient RNA interference (RNAi) was shown to increase the rate of basal and stress-induced apoptosis [37], [38]. A novel pathway for PINK1 has recently been proposed following the identification of the serine protease HtrA2/Omi as a PINK1 interactor. Mutations in HtrA2 are a susceptibility factor for PD (PARK13 locus) and loss of function models in mice display a neurodegenerative phenotype resembling parkinsonism. Phosphorylation of HtrA2 at Ser142 is dependent on PINK1 and the phosphorylated form demonstrates increased protease activity and enhanced protective effect in cells upon stress [13].

To date the majority of studies of PINK1 function come from non-mammalian models, or non-neuronal *in vitro* cell models cultured over relatively short periods of time. Whilst yielding important insights they do not recapitulate the human disease, that is, in ageing human dopaminergic neurons. To address this issue, we created a unique model of PINK1 loss-of-function, in a human neural stem cell (NSC) line that is able to differentiate into functional dopaminergic neurons [39]. This novel cellular system recapitulates the key features of PD namely dysfunction and death in ageing midbrain neurons. In addition, we utilised primary mouse neurons taken from mice and a widely used human dopaminergic neuroblastoma cell line (SHSY5Y), which allowed cross-validation in three complementary cellular systems. We report the spatio-temporal expression of PINK1 in human dopaminergic neurons, and the effects of PINK1 deficiency including chronic mitochondrial dysfunction, an increase in oxidative stress, and lysosomal pathology. These phenotypic effects may all contribute to the marked decrease in long term viability of PINK1 deficient neurons and enhanced basal and stress-induced caspase-3 activation leading to apoptosis. Our findings give new insights into the underlying dysfunction of human dopaminergic neurons in PINK1 parkinsonism and sporadic PD, by utilising a truly representative *in vitro* cellular model of the disease.

## Materials and Methods

All chemicals and reagents were obtained from Sigma or Invitrogen unless otherwise stated.

### Generation of PINK1-deficient mice

The PINK1 deficient mice were generated by Lexicon Genetics Inc. (The Woodlands, Texas, USA). **See supplementary Methods S1 online**.

### Cell culture

Human NSCs (ReNcell VM NSCs) were provided by ReNeuron Ltd. Derivation of the line from human fetal ventral mesencephalon and maintenance has previously been described [39]. Briefly, NSCs were grown in laminin coated flasks/plates (Trevigen; 20 µg/ml in DMEM:F-12) in B27 medium (DMEM:F-12 with 1X B27 supplement, 2mM Glutamine, 10 Units/ml heparin and 50 mg/ml gentamycin) containing growth factors human bFGF [10ng/ml, Peprotech] and human EGF [20ng/ml, Peprotech]. Media on NSCs was changed every 48 hours. Differentiation was carried out using either the stdD or preD method [39]. For StdD differentiation, NSCs were seeded at 30,000 cells/cm on laminin and expanded to 80% confluency in B27 medium containing growth factors (+GFs). Differentiation was initiated by changing to Differentiation medium: B27 medium without growth factors or antibiotics and supplemented with 1mM dibutyrl-cAMP (Calbiochem) and 2ng/ml GDNF (Peprotech). Media was not changed for the first 5 days, then after that every 3–4 days. For the PreD method, NSCs were trypsinised and plated out at 30,000 cells/cm onto uncoated plates in B27 medium+GFs. NSCs were left to form aggregates for 7 days without a media change. Aggregates were then transferred to laminin-coated plates of equivalent surface area in B27 medium+GFs for 3–4 days to allow cells to disperse and reach 90% confluency. Differentiation was initiated and continued as for StdD protocol. ReNcell VM NSCs are referred to as human NSCs and ReNcell VM differentiated neurons are referred to as human midbrain neurons. SHSY5Y human neuroblastoma cells were maintained as described (Muqit et al 2006).

For primary mouse cortical cultures, embryos were taken at gestational stage E16–E17. Animal husbandry and experimental procedures were performed in full compliance with the United Kingdom Animal (Scientific Procedures) Act of 1986. Cultures were set up as described in Supplementary Methods S1. Neurons were cultured in 96 well plates in maintenance medium [Neurobasal medium, 2% (v/v) B27 supplement, 2 mM glutamine, 100 I.U./ml penicillin and 100 I.U./ml streptomycin and 0.45% (v/v) D−(+) glucose]. Tissue harvested for biochemistry was snap frozen on dry ice and stored at −70°C. Embryos were obtained by crossing two heterozygote animals resulting in a mixture of F1 genotypes; genotyping is described in Supplementary Methods S1.

### Apoptosis and cell death/viability Assays

#### Annexin V PE binding

For analysis of apoptosis in NSCs and SHSY5Y cells, FACS was used to determine AnnexinV-FITC binding in conjunction with 7-Amino actiniomycin (7AAD) uptake according to manufacturer's instructions (BD Pharmingen). Briefly SHSY5Y cells were seeded at 600,000 cells per well of a 6 well plate, in triplicate, and incubated overnight in SHSY5Y medium containing DMSO or 60 nM STS (Calbiochem). Human NSCs were seeded in triplicate in 6 well plates at 200,000 per well. The following day NSCs were incubated in media containing DMSO only (1∶1000) or STS (50 nM) for 3 h at 37°C prior to processing. A total of 10,000 cells were analysed for cell death by FACS Calibur (Beckton Dickenson, CA) with the Cell Quest software.

#### Multiparameter Cytoxicity Assay

We used the Multiparameter Cytotoxicity 1 HCS Reagent kit (Cellomics) for the assessment of overall culture viability using multiple cell parameters in differentiated human and mouse neurons at different time points. The assay was carried out according to the manufacturer's instructions. Plates were analysed with an ArrayScan© HCS reader and Cytotoxicity Indices (CIs) calculated using the Multiparameter Cytotoxicity 1 BioApplication Software (Cellomics). A full explanation of this algorithm is provided on the supplier's website: http://www.cellomics.com/content/menu/MP_Cytotoxicity/. Briefly, nuclear size, morphology and cell density measurements are made on nuclei automatically identified in fluorescence channel 1. The BioApplication then measures nuclear intensity in channel 2 and the intensity of lysosomal stain in the cytoplasmic region in channel 3. Upper and lower thresholds are automatically set for nuclear morphology/size, cell membrane permeability and lysosomal mass from user-configurable reference wells (control or wild type neurons). The percentage (indices) of cells in each well that are outside of these thresholds is ascertained. The Cytotoxicity Index is the maximal of nuclear morphology index, membrane permeability index, lysosomal mass index and cell density index, for each well. A mean CI is calculated based on three replicate wells per clone/genotype.

#### Counting apoptotic nuclei

Human neurons were differentiated using the Pre-D protocol on poly-L-lysine glass coverslips (Biocoat) pre-coated in laminin. At 5 and 43 days differentiation (dd), cultures were incubated with either 1 mM STS or vehicle (DMSO) for 3 h before being fixed. Immunostaining was undertaken using dual labeling with anti-βIII tubulin, anti-TH and Hoechst 33342 as described [39]. Images were taken using an Axioplan Imaging microscope (Zeiss) using a 25X objective (see below). At least 50 adherent neurons per culture were examined and their nuclei scored as normal or pyknotic (fragmented/condensed) by a blinded observer. In addition, images from 5–10 non-overlapping fields of view were taken per culture using Axioplan software. Images were printed and the percentage of pyknotic versus normal neuronal nuclei were scored.

#### Cytochrome C release

Release of cytochrome c from mitochondria in SHSY5Y cells was carried out using the Apoptosis Detection Kit (MitoSciences) according to manufacturer's instructions.

### SDS-PAGE and immunoblot analysis

For details see **supplementary Methods S1 online**. Cells were lysed by the direct addition of sample buffer and protein lysates separated on 10% or 16% Tris-Glycine mini gels by SDS-PAGE, before transfer onto nitrocellulose membranes. Membranes were incubated in non-fat milk in PBS with 0.1% Tween-20 (PBST) then with primary antibodies (see **Table S1**). Membranes were washed then incubated with HRP-conjugated secondary antibodies (Dako). Membranes were incubated with SuperSignal West Pico Chemiluminescent Substrate (Pierce) and bands detected with x-ray films (BioMax). Equivalent protein loading was confirmed by stripping membranes and re-probing with anti-β-actin antibody. Band densitometry was performed using Quantity One 4.6.6 software (BioRad).

### RNA extraction, cDNA synthesis and RT-PCR

Total RNA was isolated using TRIzol reagent and cDNA synthesised using random primers according to manufacturer's instructions. cDNA was diluted 1∶5 in water prior to PCR. All TaqMan Real Time PCR reactions were performed using using FAM-labelled PINK1 probe and VIC/TAMRA-dye labelled Endogenous control Human RPLPO or GAPDH (Applied Biosystems). PCR was performed using the ABI PRISM 7700 Sequence Detection System. Gene expression was calculated using the 2^-δδCT^ method [40].

### Synthesis and packaging of retroviral constructs

Optimal short hairpin RNA 19mer sequences targeted to human PINK1 (NM_032409) were determined using the Dharmacon siDESIGN Center (http://www.dharmacon.com). The 19mers with the highest predicted efficacy (scores ≥7) were selected. Single stranded 64mers were obtained from Operon. The forward and reverse sequences are detailed in supplementary Methods S1 online. Oligomers were annealed and ligated into pSUPER.retro.puro (PSR) mammalian expression vector (Oligoengine) according to the manufacturer's protocol. Ligation mixtures were used to transform JS4 competent *E.coli.* Maxipreps of successfully ligated vectors were prepared using a Quaigen kit and sequenced. Vectors were packaged into retroviral particles using ψ-NX-Ampho cells. Viral supernatant harvested from transfected cells was sterile filtered and stored at -70°C until required.

### Production of stable cell lines

Early passage NSCs were infected with viral supernatant containing PSR vector, PSR containing shRNA sequences (supplementary Methods S1 online). When confluent, NSCs were split and transduced cells selected for using Puromycin (1 ug/ml). Cell colonies were picked and further propagated before screening by RT-PCR.

### Immunofluorescence and image acquisition

Immunostaining of coverslips and 96 well plates was carried out as described previously [39]. Details of all antibodies are available in supplementary data online (Table S1). For live dye staining of organelles, Mitotracker Red CMXRos or Lysotracker was used according to manufacturer's instructions. Coverslips were mounted onto slides using fluorescent mounting medium (Dakocytomation). Non-confocal images were obtained using an Axioplan 2 MOT microscope (Zeiss) with filters for FITC, Rhodamine and DAPI and Plan Neofluar 25x/0.8 Ph2 objective at RT. Images were taken using an AxioCam MRm (Zeiss) camera and controlled using the Axiovision Control software. Alternatively, images were obtained using an LSM510 META confocal microscopy system (Zeiss) equipped with “plan-Apochromat” 63x/1.40 Oil DIC objective at RT controlled by Zeiss LSM software. Fluorescence was recorded at 488nm using 30mW Ar-laser for excitation or at 543 nm using 1mW HeNE laser for excitation. Zeiss Immersol was used as imaging medium. For counting of tyrosine hydroxylase (TH) and neurofilament (NF) positive cells, the Cellomics ArrayScan© HCS reader was used and images from 30 fields of view per well were analysed using the Target Activation BioApplication software.

### Transmission Electron Microscopy

Human NSCs were plated on 13mm Thermanox coverslips coated with laminin. When confluent, NSCs were differentiated using the SD/DA protocol for 47 dd and fixed using 3% glutaraldehyde in 0.1 M sodium cacodylate buffer and 5 mM CaCl_2_, pH 7.4. Specimens were dehydrated through a graded series of Analar Ethanol (70% 90% 100%). Cells were embedded in araldite resin mixture and sectioned using a Richert Ultracut S microtome. Sections were stained using 25% Uranyl acetate in methanol and Reynold's Lead Citrate and images were taken on a Philips CM10 transmission electron microscope at 3800X and 9800X magnification. Images were taken using the KeenView system from SIS, resized and annotated in Adobe Photoshop.

### Live cell imaging

Live cell imaging was performed as described previously [41]. See **supplementary Methods S1 online.** Briefly, cells were bathed at RT in a HEPES-buffered salt solution (HBSS) containing (mM): 156 NaCl, 3 KCl, 2MgSO_4_, 1.25 KH_2_PO_4_, 2 CaCl_2_, 10 glucose and 10 HEPES, pH 7.35. For Δψm measurements, 25 nM TMRM was included and cells were incubated for 45 min. Z-stacks of >20 neurons per clone were obtained using a Zeiss LSM confocal microscope. Images were analysed using Lucida 6 software. For ROS measurements, MitoSOX (10 µM) was loaded for 10 min followed by washing. Dihydroethidium (HEt, 10 µM) was present in all solutions throughout the experiments. Confocal images were obtained using a Zeiss 510 uv-vis CLSM equipped with a META detection system and a 40x oil immersion objective. HEt or MitoSOX were excited using the 543 nm laser line and fluorescence measured using a 560nm longpass filter.

**Measurement of total and reduced glutathione.**

See **supplementary Methods S1 online**

Citrate synthase activities

### Statistical analyses

All experiments were performed with three different cell clones, in triplicate. Data were analysed by parametric Student T-tests and significance expressed as follows *P<0.05, **P<0.01, ***P<0.001 unless otherwise stated. For all graphs bars represent mean±SEM.

### Online supplementary material

Generation of PINK1 knockout mice

Primary embryonic mouse cultures

Table S1 Antibodies

SDS-PAGE and immunoblotting

Production of stable cell lines

Measurement of total and reduced glutathione

Live cell imaging

ETC activities

## Results

### PINK1 is up-regulated following differentiation of human NSCs to dopaminergic neurons

**Figure 1:**
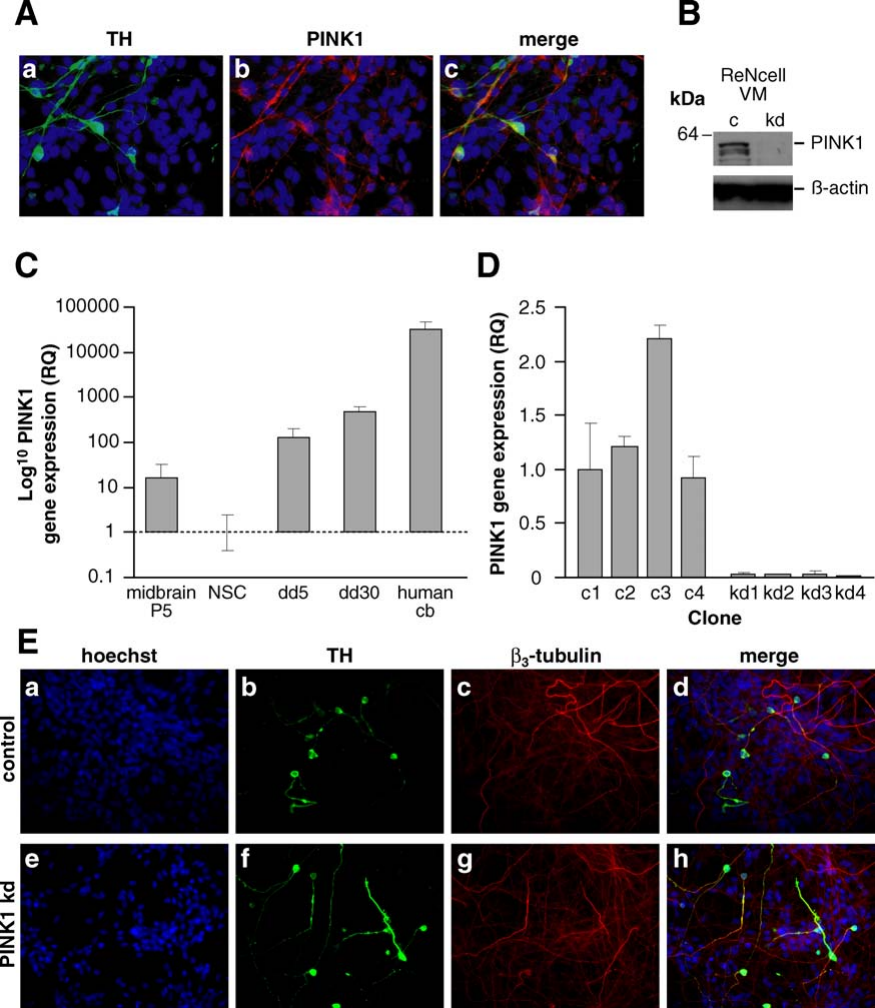
PINK1 Expression & Up-regulation. A) PINK1 expression in human dopaminergic neurons. Red; PINK1, green; TH, blue; hoescht. B) Western blot showing endogenous PINK1 protein expression in control (c) and PINK1 kd (with 90% mRNA knockdown) human neuronal lysates after 5 days differentiation. Anti-PINK1 (Novus) was used to detect PINK expression; β-actin levels are shown as loading control. C) Histogram showing relative fold change in PINK1 mRNA expression levels between NSCs, 5 day differentiation (dd5), 30 day differentiation (dd30). PINK1 expression levels from pre-immortalised neural stem cells from 8 week old ventral mesencephalon (Midbrain P5) and adult human brain (Human Cb) are shown for comparison. Values represent mean of three replicate samples±s.e.m. D) Histogram showing expression of PINK1 mRNA in human neurons in four control (c1-4) and four PINK1 kd (kd1-4) selected clones relative to an endogenous control gene. RQs were normalised to that of c1; values represent mean of 3 replicate samples±standard error (s.e.m). E) Immunofluorescence images showing dopaminergic cells in control (i) and PINK1 kd (ii) human neuronal cultures after 5 days differentiation. Red; TH, green; βIII tubulin, blue; Hoecsht.

We first sought to validate the use of the human NSC model, differentiated to human midbrain neurons, as a useful model to test PINK1 function. RT-PCR confirmed upregulation of expression of a range of dopaminergic neuronal markers (tyrosine hydroxylase, TH, dopamine transporter, DAT) upon differentiation of NSCs to human neurons (**Figure S1A**). In addition there was an upregulation of the expression of markers Lmx1a and Nurr1, both of which are involved in committing precursor cells to dopaminergic differentiation. Furthermore we demonstrated that the human neurons expressing TH, also expressed PINK1. PINK1 expression is highest in mature neurons as demonstrated by immunofluorescence (**Figure 1A**). PINK1 was detected by RT-PCR in human NSCs (**Figure 1C**). Moreover, PINK1 expression was found to increase up to ∼100 fold following differentiation to neurons, (**Figure 1C**) suggesting a physiological role of PINK1 within differentiated human neurons. This high level of PINK1 expression in differentiated human DAergic neurons validated the use of this model in examining the effects of loss of PINK1 function.

### RNAi-mediated knockdown of PINK1 mRNA in human cell lines

Using retroviral mediated PINK1 shRNA expression, levels of PINK1 knockdown ranging from 10–90% were achieved. The efficiency of PINK1 knockdown (PINK1 kd), determined by RT-PCR, was found to be shRNA sequence specific, with sequence #1029 resulting in >90% kd consistently in both the SHSY5Y and the human NSCs. (**Figure S1 B**). shRNA directed against a different gene (Lamin AC) did not reduce PINK1 mRNA levels (see S1B), confirming that this method did not result in non-specific PINK1 kd. Results shown are normalized for housekeeping gene, LRPO. However RT_PCR for other genes including two other control genes (β-actin and GAPDH) as well as other PD genes (parkin and DJ-1) did not show significant alterations in expression between control and PINK1 kd (data not shown).. Knockdown of endogenous PINK1 mRNA was maintained after differentiation as human neurons (**Figure 1D**) even taking into account the ∼100 fold increase in PINK1 expression described above. Analysis of protein levels by Western blotting confirmed reduced levels of protein expression in #1029 clones compared with controls (**Figure 1B**). We similarly confirmed absence of PINK1 expression in cultured cortical neurons from the PINK1 knockout mouse model by Western blot (**Figure S1D**).

### PINK1 is not necessary for differentiation of human neural stem cells to dopaminergic neurons

Human NSC lines, carrying the stable #1029 PINK1 kd shRNA, does not prevent differentation into human DAergic neurons as demonstrated by tyrosine hydroxylase (TH) immunostaining, following the PreD differentiation protocol (**Figure 1E**). No difference in TH expression was found between PINK1 kd neurons and controls using Western blot (data not shown). The PreD protocol generates the highest proportion of DAergic neurons in ReNcell cultures [39]. However, due to the formation of ‘neuronal aggregates’ in these cultures, accurate quantification of percentage TH+ neurons is extremely difficult using microscopy. In contrast, the StdD protocol gives rise to a monolayer of neurons, which permits the use of the Cellomics microscope and BioApplication software to robotically count (cytoplasmic) TH+ neurons. Control or PINK1 kd human neuronal cultures contained 1–5% TH+ neurons using the StdD protocol. There was no significant difference in the proportion of TH+ cells in control or PINK1 kd cells, either as a percentage of total cells or percentage of total neurons (data not shown).

### PINK1 deficiency results in age-related reduction in basal viability of human and mouse neurons

**Figure 2:**
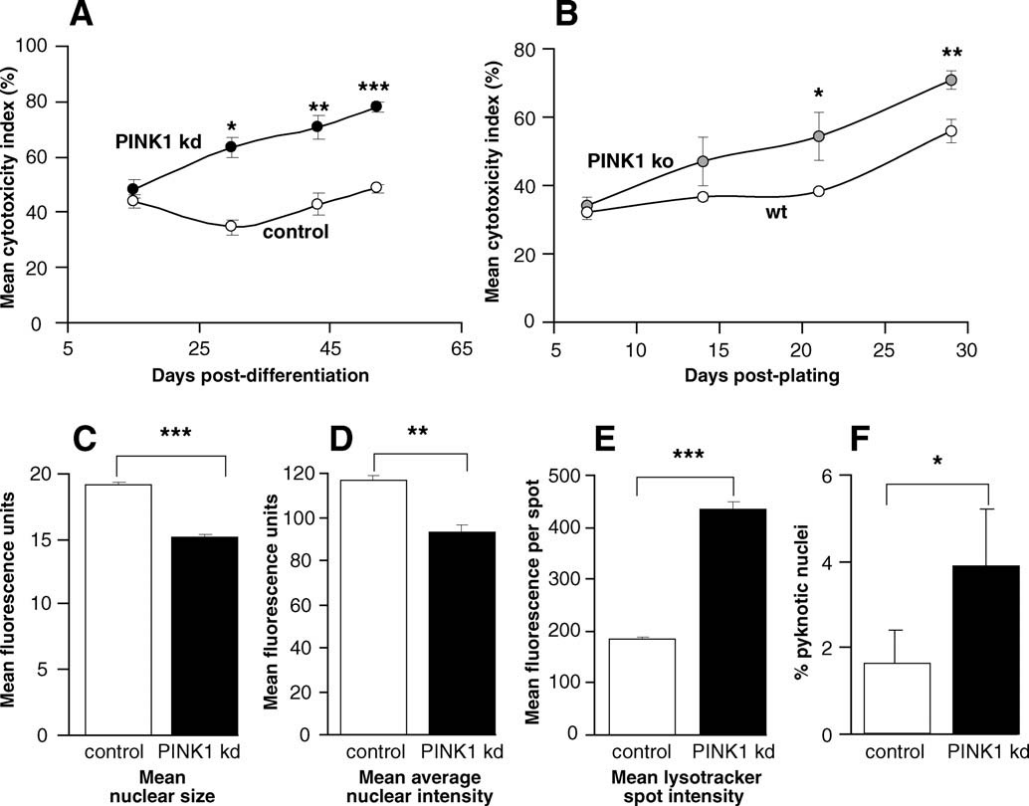
Long term viability is reduced in PINK1 deficient neurons. A) Graph showing an increase in mean CI over time of human PINK1 kd neurons compared to controls. Values shown are means±s.e.m of 3 control and 4 kd cultures, each measured in triplicate. B) Graph showing an increase in mean CI over time of mouse embryonic cortical neuronal cultures taken from wild type and PINK1 knockout mice. Values shown are means±s.e.m of wild type (PINK1+/+, n = 2), and homozygote (PINK1−/−, n = 3) cultures each measured in triplicate. C,D,E) Comparison of individual cell parameters assayed by the Cytotoxicity algorithm for aged human neurons (dd 52) with PINK1 kd compared to controls, including mean nuclear size, mean nuclear intensity, plasma membrane permeability and lysosomal mass/pH. Values shown are means±s.e.m of 3 control and 4 kd cultures, each measured in triplicate. F) Histogram showing percentage apoptotic neuronal nuclei in aged control and PINK1 kd human neurons under basal conditions. Values shown are mean percentages of neuronal nuclei that are pyknotic from 10 fields of view, ±sem from 3 independent cultures.

We then investigated if PINK1 was necessary for the survival of neurons. Due to the characteristics of the different cell models, we employed different methods to assess viability and apoptosis. SHSY5Y cells are proliferative neuroblastoma cells that may be assessed using flow cytometry for both viability and apoptosis. Annexin V/PI flow cytometry of PINK1 kd SHSY5Y cells showed a significant reduction in the number of live cells after 96 h in culture when compared to control cells (p = 0.0263). Furthermore we observed a concomitant increase in cells undergoing early apoptosis, as shown by cells expressing annexin V only (p = 0.0023), and cells staining for both PI and annexin V (p = 0.0218) (**Figure S2B**). To assess the viability of differentiated human neurons, we employed a cytotoxicity assay for neurons at days 15, 30, 43 and 52 in culture. Cytotoxicity Indices (CI) for control and PINK kd cultures were calculated at each time point. There was no significant difference in basal viability at day 15, but from days 30 to 52, there was a significant and increasing difference in the mean CI of PINK1 kd neurons compared to controls, demonstrating an age-dependent effect of loss-of-PINK1 function (**Figure 2A** **)**. By 52 days differentiation, the mean CI of PINK1 kd neurons was ∼30% above that of controls (*p<*0.001). Using the Cellomics software we examined differences in the various parameters quantified using the Multiparameter Cytotoxicity 1 BioApplication Software which are collated into the final CI readout. These revealed a striking reduction in mean nuclear size and fluorescence intensity, indicative of apoptosis (**Figure 2C,D**). Indeed we found a significant increase in the percentage apoptotic neurons in aged PINK1 kd cultures compared to controls by manual counting of pyknotic nuclei (**Figure 2F**). In addition there was a significant increase in mean lysosomal mass/pH in aged PINK1 kd neurons (**Figure 2E**).

We then sought to confirm these findings using primary embryonic cortical cultures from PINK1 KO mice (**Figure S2C)**. Again we found the basal viability of neuronal cultures lacking PINK1 declined with age with mouse cortical KO neurons having significantly higher CI than controls by later time points (**Figure 2B**). Assessment of individual Multiparameter Cytotoxicity assay parameters at later time points (d30) revealed a decrease in nuclear intensity, and an increase in lysosomal mass/pH (**Figure S2Di,ii**) indicative of early cellular apoptosis.

### Lack of PINK1 sensitises neurons to mitochondrial-mediated apoptosis

**Figure 3:**
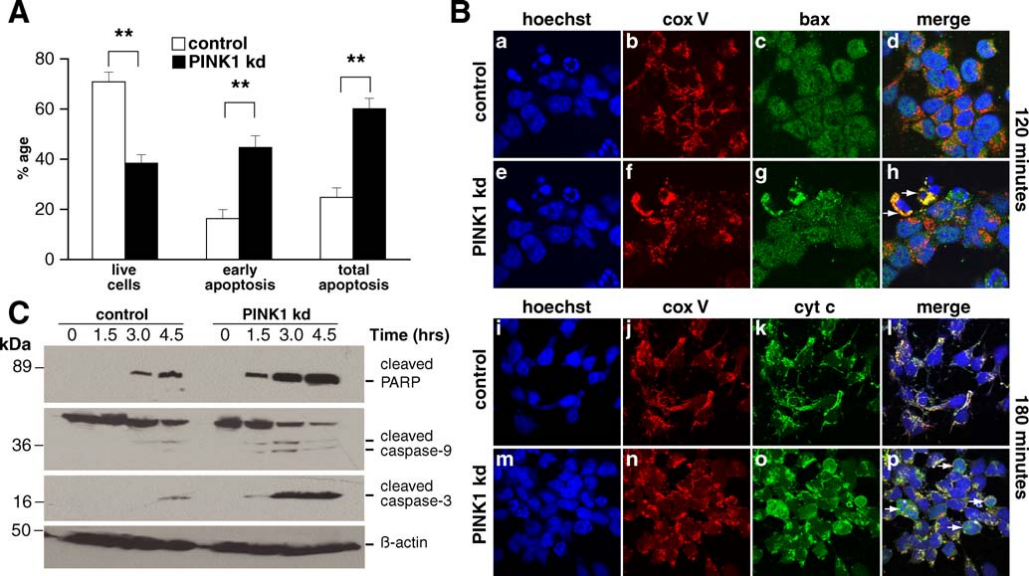
Apoptosis pathway in PINK1 kd SHSY5Y cells. A) Histogram showing a significant increase in apoptosis in PINK1 kd SHSY5Y cells in response to STS compared to controls. Cells were incubated with 60nM STS or vehicle overnight prior to analysis of annexinV/PI binding by FACS. Values represent mean of 20,000cells/well, of duplicate wells of 3 clones across 5 independent experiments±s.e.m. B) Panel a-h shows double immunofluorescence using Bax and Cox V antibodies in SHSY5Y cells treated with 0.75 µM STS. At 120 minutes, Bax (green) is seen in the cytoplasm and nucleus of control cells, with no overlap with a mitochondrial marker Cox V (red). In PINK1 kd cultures, arrows highlight two cells with evidence of nuclear condensation suggestive of apoptosis. In these cells, Bax immunofluorescence overlaps with Cox V immunofluorescence indicating Bax localisation in the mitochondria. Panel l-p shows double immunofluorescence using cytochrome c and Cox V antibodies. At 180 minutes, there is overlap of cytochrome c (green) and Cox V (red) in control cells indicating cytochrome c localisation in the mitochondria. However in PINK1 kd cells arrows indicate several apoptotic cells with diffuse cytosolic cytochrome c, indicating translocation from mitochondria to cytoplasm. Images are representative of two independent experiments with 3 clones. Images were acquired by obtaining z-stacks through cells at x63 oil objective. C) Western blot showing increased apoptosis on exposure to STS in PINK1 kd SHSY5Y cells. Cells were incubated with 0.75 µM µM STS for 6hrs. Cleaved caspase 9, cleaved caspase 3, and PARP expression is increased at earlier time points in PINK1 kd cells than in controls.

Using annexin V based flow cytometry we demonstrated that SHSY5Y cells exposed to 60nM staurosporine (STS) for 24hrs exhibited a significant reduction in the number of live cells in PINK1 kd clones compared to controls (p = 0.0002), as well as higher levels of early apoptosis in PINK1 kd cells (p<0.0001), and higher levels of total apoptosis in PINK1 kd cells (p = 0.0002) (**Figure 3A**). Preincubation of PINK1 kd cultures with 100 µM of the pan-caspase inhibitor Z-VAD-FMK 1hr prior to exposure with STS abolished cell death and markers of early and late apoptosis (data not shown).

To assess the pathway of STS-induced apoptosis, and how it is sensitized in PINK1 kd cells, we used 0.75 µM STS for 4hrs, fixed cells at different intervals and performed double immunofluorescence to assess the localisation of Bax and cytochrome c. Under basal conditions Bax was observed in the cytosol and nucleus of cells, cells but not in the mitochondria (demonstrated by the failure of Bax to co-localise with the mitochondrial protein Cox V) whereas cytochrome c was localised to the mitochondria and showed overlap with Cox V staining. At 120 mins after addition of STS, some cells in the PINK1 kd cultures appeared apoptotic with nuclear fragmentation and condensation. In these cells, Bax staining co-localised with Cox V, suggesting translocation of Bax to the mitochondria from the cytosol in PINK1 kd cells undergoing apoptotic stress (**Figure 3B**,**a–h**). We further confirmed this finding using subcellular fractionation into mitochondrial and cytosolic fractions and immunoblotting for Bax (data not shown). After 180 mins of exposure to STS, we observed several cells per field in PINK1 kd cultures undergoing apoptosis as evidenced by nuclear fragmentation and condensation. In these cells Cox V staining demonstrated a loss of the tubular network of mitochondria, mitochondrial fragmentation and abnormal clustering. In addition to these nuclear and mitochondrial abnormalities, cytochrome c was distributed diffusely in the cytoplasm of these cells, confirming that it had been released from the mitochondria during apoptosis (**Figure 3B**, **i–p**).

To further confirm the induction of apoptosis in PINK1 kd cells, we assessed PINK1 kd cell lysates for active caspase-3 and PARP cleavage at 90 minute intervals following exposure to 0.75 µM STS. We demonstrate an increase in cleaved caspase 3, caspase 9, and an increase in cleaved PARP in PINK1 kd cultures compared to controls (**Figure 3C**). All these molecular events were activated at earlier time points in the PINK1 kd cells compared to controls suggesting an enhanced susceptibility to apoptosis via the mitochondrial pathway.

**Figure 4:**
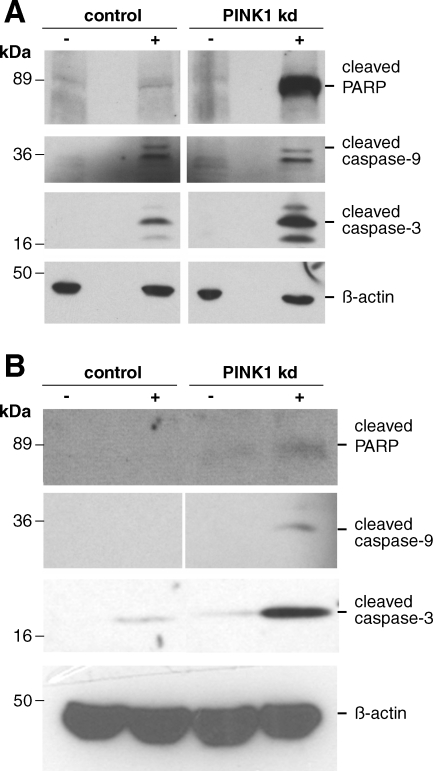
Apoptosis in PINK1 kd human neurons. Western blots showing increase in basal (−) and STS-induced (+) cleaved caspase 3, cleaved caspase-9 (two bands ∼36 and ∼32 kDa) and cleaved PARP without PINK1 in young (A) (dd5) and aged (B) (dd43) human control and PINK1 kd neuronal cultures. Each lane is a sample from three pooled independent culture lysates. The lowest bands are immunoreactive degradation products which run at the foot of an SDS-PAGE gel.

We investigated whether a similar sensitivity to mitochondrial apoptosis was evident in the human neurons as well as the SHSY5Y cells. Both young and aged human PINK1 kd neurons demonstrated enhanced sensitivity to STS and a higher induction of mitochondrial apoptosis with increased expression of cleaved caspase 3, caspase 9, and cleaved PARP (**Figure 4** **A, B)**. The increased expression was confirmed using band densitometry. Of note, the immunoreactive bands obtained using lysates from d43 neurons are less intense than those of young neurons due to the reduced amount of material obtained from aged cultures. Interestingly, activated caspase-3 was also detected in lysate from PINK1 kd neurons treated with vehicle alone at d43 (**Figure 4** **B)** suggesting an enhanced basal level of caspase-3-mediated apoptosis in the absence of PINK1 in aged neurons.

### PINK1 deficiency in neurons leads to mitochondrial morphometric abnormalities

**Figure 5:**
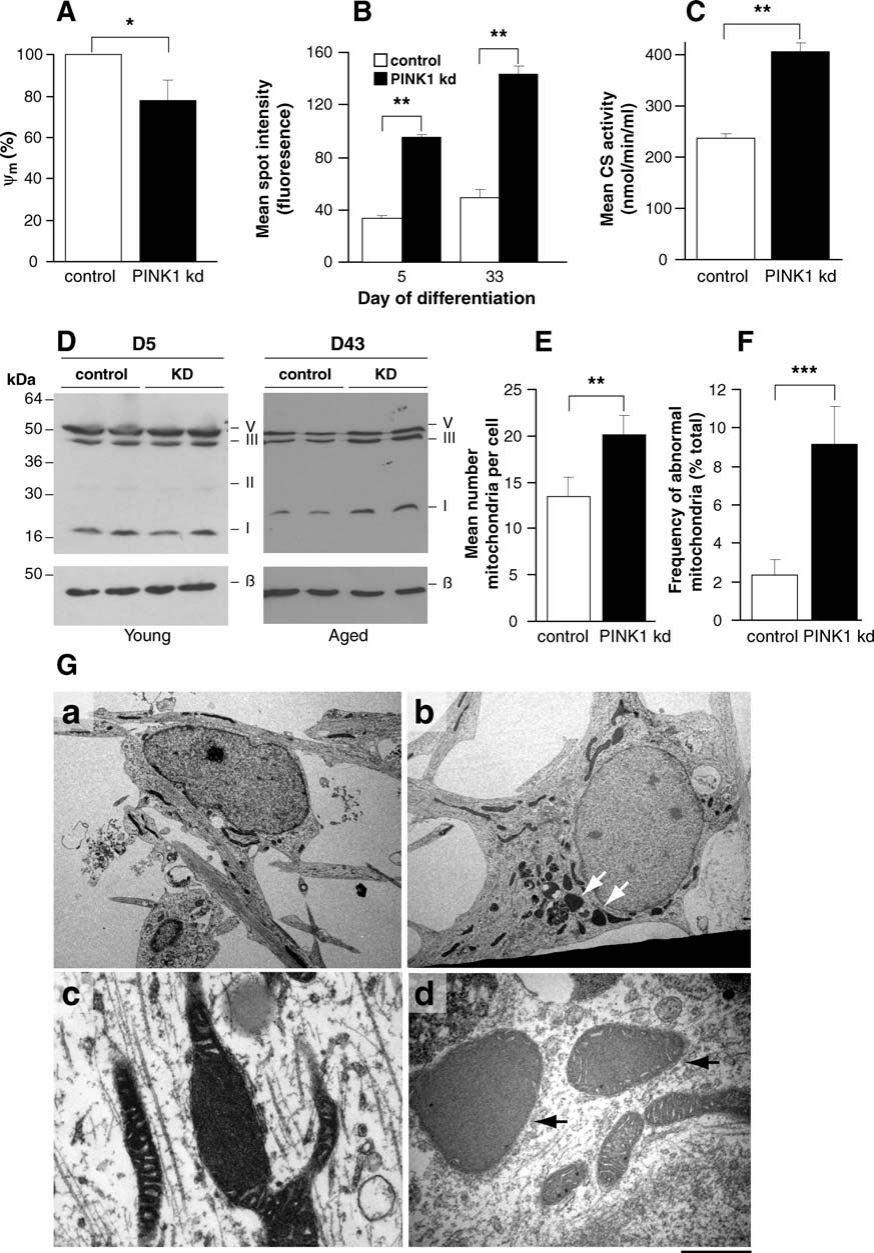
Mitochondrial abnormalities in PINK1 deficient neurons. A) PINK deficiency leads to a reduced basal mitochondrial membrane potential (ψm) in young PINK1 kd neurons compared to controls, analysed using TMRM fluorescence. Values are mean percentages of the control cultures (normalised to 100%) from three independent experiments. B) Histogram showing increased uptake of the redox sensitive dye MitotrackerRed in human PINK1 kd neuronal cultures compared to controls at early and later time points. Values represent means±sem of 3 control or three PINK1 kd cultures (measured in triplicate). C) Histogram showing increased citrate synthase (CS) activity in aged (d30) PINK1 kd human neurons compared to controls. Values represent means±sem from three independent cultures. D) Western blot showing expression of mitochondrial subunits in young (D5) and aged (D43) human neurons lacking PINK1 compared to controls. Note the increase in complex subunit expression in aged neurons lacking PINK1. Replicate lanes of pooled samples from three independent cultures are shown. Expression of OXPHOS subunits C-I-20 (ND6), C-II-30 (FeS), C-III-Core-2 and C-V-α is shown. β-actin; loading control. E) Graph showing significantly increased mean number of mitochondria/cell in aged PINK1 kd human neurons compared to controls, as quantified by TEM analysis. Histogram shows mean number mitochondria per cell ±sem averaged from at least 50 cells. F) Increased frequency of abnormal mitochondria of aged human PINK1 kd neurons compared to controls, quantified using TEM. Histogram shows mean frequency of abnormal mitochondria (per total number mitochondria in each cell) ±sem, n = 50 cells. G) TEM images showing abnormal mitochondria (arrowheads) within aged (d47) human neurons lacking PINK1 (panel b, and at higher magnification in d) compared to control neurons (panel a). There are increased numbers of mitochondria within cells lacking PINK1 (panel b) compared to controls (panel A) and a higher proportion of mitochondria in PINK1 kd neurons appear swollen with disorganised christae (panels c, d). Scale bar in a,b; 10 µM, c  =  500 nM.

We investigated mitochondrial dysfunction in PINK1 kd cells using live cell imaging of TMRM fluorescence. We found that PINK1 deficiency leads to a reduction in mitochondrial membrane potential (ψm) in both SHSY5Y cells (data not shown) and human neurons (**Figure 5A**). There was a mean reduction of 22% in the TMRM signal in PINK1 kd compared to controls (p = 0.046)(n = 60 neurons/culture taken from 3 individual clones). In addition, we found several lines of evidence suggesting increased mitochondrial proliferation in both young and aged human PINK1 kd neurons and in cortical PINK1 ko mouse neurons. First, with human PINK1 kd neurons, we found an increased uptake of the redox-sensitive dye, Mitotracker CMXROS, suggesting an increase in mitochondrial mass compared to controls (**Figure 5B**). Second, in PINK1 deficient neurons we found a marked up-regulation of mitochondrial OXPHOS complex subunits in aged human neurons as analysed by Western blot (**Figure 5D**). There was no significant percentage change in subunit expression between PINK1 kd and control neurons at dd5. However, by dd43, expression of complex I, III and V subunits had significantly increased in PINK1 kd neurons by 107.8±22.5 %, 125.1±5.4% and 233.8±17.9% respectively, as determined by band densitometry (p<0.01). Third, *in vitro* assays showed an increase in the mitochondrial citrate synthase activity within these cells (**Figure 5C**). We also found a significant increase in mitochondrial mass using direct quantification of mitochondria within individual neurons using transmission electron microscopy (TEM) (**Figure 5E**). However, no parallel increase in respiratory complex activity was detected using biochemical techniques in neuronal models (data not shown). Together these data suggest a compensatory increase in mitochondrial density in neurons lacking PINK-1. TEM analysis of young and aged human neurons lacking PINK1 also revealed an increase in the proportion of abnormal swollen mitochondria within cells (**Figure 5F,G**) further supporting the hypothesis that PINK1 functions to maintain mitochondrial integrity in neurons.

### PINK1 deficiency in neurons is associated with an increase in basal free radical production and decreased steady state levels of glutathione

**Figure 6:**
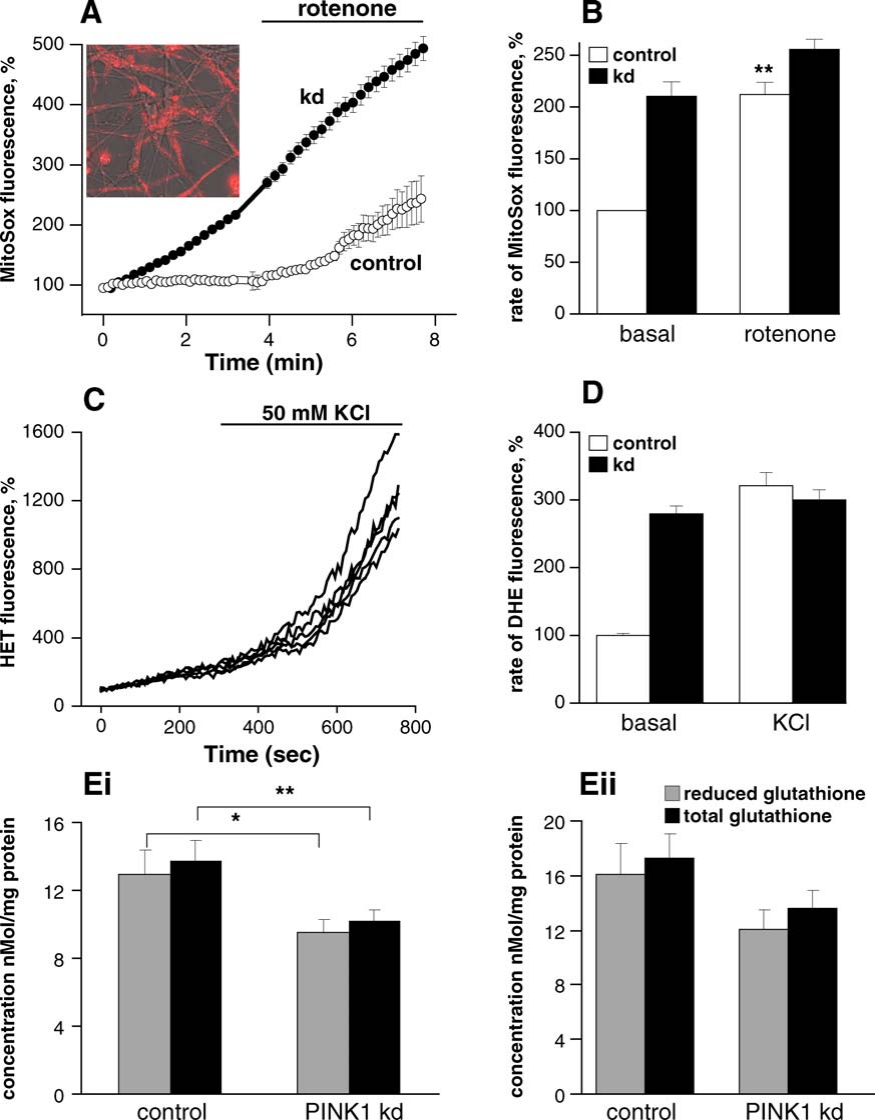
Free Radical Production in PINK1 knockdown neurons. A) and (B) demonstrate a higher basal production of ROS production in mitochondria of PINK1 kd neurons compared to controls. Image in Figure 6A confirms mitochondrial localisation of Mitosox probe in neurons. Inhibition of complex 1 with rotenone induced a significant increase of ROS production in control cells. Inhibition of complex 1 in PINK1 kd cells slightly increased ROS production which is still higher than rotenone induced ROS production in control mitochondria. The basal rate of Mitosox fluorescence in control cells was taken as 100%. C) Shows an increase in the rate of HEt fluorescence in response to 50 mM KCL in control neurons. D) PINK1 kd neurons have higher basal rates of cytosolic ROS production compared to control neurons. Stimulation using KCl results in an increase in ROS production which is more marked in control than PINK1 kd neurons. E) Histogram showing decreased reduced and total glutathione concentrations in young (dd5 i) and in aged (dd43 ii) and human PINK1 kd neurons compared to controls. Values represent means ±sem from triplicates of three independent samples.

We utilised live cell imaging techniques using the redox-sensitive dye dihydroethidium (HEt) which measures cytosolic ROS production and the mitochondrial targeted variant of this dye, Mitosox, to measure mitochondrial ROS production. (Figure 6A,B) The basal rate of ROS generation was significantly increased in the cytoplasm of PINK1 kd human neurons, showing a 2.79-fold increase in basal rate of fluorescence increase (mean = 279±11.3%, n = 189 cells, p<0.001). Stimulation of neurons with 50 mM KCl to transiently raise [Ca^2+^]_c_, increased the rate of ROS production in control cells, which showed a 3.2 fold increase in the rate of HEt fluorescence (from basal 100% to 321±19.3% while the response in PINK1 kd neurons was much smaller (from 279±11.3% to 300.1±14.7%, where 100%- basal rate of ROS in control neurons) (**Figure 6** **C,D**). We demonstrated a higher basal rate of mitochondrial superoxide production in PINK1 kd neurons compared to controls (the rate of increase of MitoSOX fluorescence was 2.1-fold higher in PINK1 kd neurons (mean = 212±14.1%, p<0.001). The complex I inhibitor, rotenone caused a smaller proportional increase in superoxide production in PINK1 kd (210.2% to 255.4±9.9%) compared to controls (100% to 212.1±11.6%) due to the higher basal levels of ROS generation, although the absolute rate of ROS generation in response to rotenone was higher in PINK1 kd neurons. (**Figure 6A,B**)

In addition to measuring ROS production we assayed the antioxidant defense mechanisms by analysing glutathione levels in young (dd5) and aged (dd43) human neurons. (**Figure 6E** **i, ii**). We demonstrate a significant reduction in the *total* levels of glutathione (reduced+oxidised) in PINK1 kd cells, implying an impairment of glutathione synthesis. A decrease in the ratio of reduced:oxidised glutathione was not found.

### Aged midbrain derived neurons deficient in PINK1 contain lysosomal aggregates

**Figure 7:**
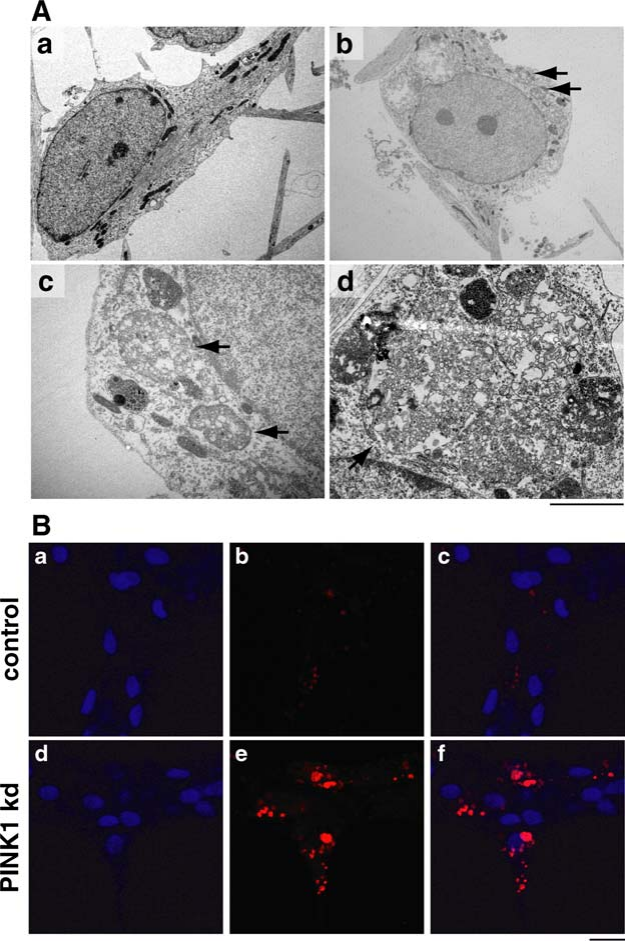
Abnormal lysosomal morphology in PINK1 kd neurons. A) TEM images of vesicular aggregates within surviving aged human neurons lacking PINK1 at dd47. A control neuron is shown in panel A. Panels b-d show aggregates in PINK1 kd neurons, panel c is an aggregate enlarged from b. Scale bars; a, b; 20 µM, d; 2 µM. B) Immunofluorescence images of aged (dd47) control human neurons (panels a-c) and PINK1 kd neurons (d–f) showing lysosomal morphology (red staining; panels b and e). Nuclei are shown in blue (panels a and d), merged images presented in panels c and f. Large and multiple aggregates seen only in PINK1 kd neurons are positive for the dye Lysotracker (panel d). ,Scale bar  =  20 µM

In addition to morphological mitochondrial abnormalities in aged human PINK1 kd neurons, we also found large intracytoplasmic vesicular aggregates only in PINK1 kd neurons, reminiscent of autophagosomes, at a frequency of approximately 30% (**Figure 7A**). To determine whether these structures were lysosomal in origin, we applied the acidophilic Lysotracker dye to aged cultures and found this dye was taken up into these distinctive aggregates, which were considerably larger and more numerous in PINK1 kd neurons compared to controls (**Figure 7B**).

The phenotypes observed in the different cell models of PINK1 loss-of-function are summarised in Table 1.

**Table 1:** Summary of the phenotypes observed with PINK1 deficiency in the different cell models used.

Model	PINK1 deficiency	Reduced viability	Sensitivity to apoptosis	Mitochondria dysfunction	Oxidative stress	Lysosomal dysfunction
SHSY5Y human	shRNA	Age-related	Basal STS-induced	↓Δψ_m_	N/A	N/A
Midbrain neurons human	shRNA	Age-related	Basal STS-induced	↓Δψ_m_ Altered mitochondrial morphology	↑ROS ↓ total glutathione	↑autophago-lysosomes ↑ lysotracker uptake
Cortical neurons mouse	Trans-genic	Age-related	Basal	N/A	N/A	↑ lysotracker uptake

N/A  =  not assessed

## Discussion

The aim of this study was to further elucidate the function of PINK1 in the prevention of DAergic degeneration seen in Parkinson's disease. We produced stable knockdown models of PINK1 using small interfering RNAs in two complementary cell lines–human neuroblastoma SHSY5Y and a novel human NSC line capable of high levels of DAergic differentiation. In addition, we used primary neuronal cultures from PINK1 knockout mice to corroborate our findings and control for potential non-specific effects of RNAi. Human neurons are generated from immortalised human fetal ventral mesencephalic neural stem cells (NSCs) which maintain a stable karyotype in culture, stable growth rates and readily differentiate into function neurons–thus providing advantages over commonly used “cancer” cell lines [39]. Differentiated NSCs cultures contain a high percentage of DAergic neurons which express typical neuronal markers and display characteristic action potentials. The use of stable RNAi allowed us to study the molecular changes occurring in human DAergic neurons during the process of ageing in a PINK1 null background. Our model therefore represents an *in vitro* system highly relevant to human PD, which is both progressive and age-dependent.

### PINK1 expression in dopaminergic neurons

We found that PINK1 expression is up-regulated ∼100 fold during differentiation of human NSCs into human neurons, suggesting that PINK1 has an important function in differentiated neurons. However, it is important to note that PINK1 did not affect differentiation into the DAergic phenotype. This suggests PINK1 is not necessary for development which is in-line with the progression of the PINK1 mutation onset of PD. Endogenous PINK1 was expressed within soma and neurites of post-mitotic dopaminergic neurons, although it was not expressed exclusively in these subtypes. This is consistent with previous reports which show endogenous PINK1 expression in both neurons and glia throughout brain [21], [24]. PINK1 expression in neurons was highest within mature processes, where its expression may be most critical.

### PINK1 deficiency and long term survival

A major finding in our study was the reduction in long term viability of human and mouse neurons deficient in PINK1. To our knowledge this is the first age-dependent phenotype reported for any *in vitro* cell model of PD. A progressive phenotype has been reported in PINK1 knockout *Drosophila* models, attributable to an increased level of apoptosis, but so far not in mammalian models [32], [34]. We observed significant *age-dependent* increases in cell death and apoptosis in mammalian neurons which provide a mechanism for the progressive phenotype seen in both sporadic and genetic forms of PD which have up until now been difficult to recapitulate *in vitro*.

### PINK1deficiency sensitises neurons to mitochondrial apoptosis

An increase in apoptosis has been previously reported in SHSY5Y cells following transient RNAi-mediated PINK1 kd, and in fibroblasts taken from patients with compound heterozygous PINK1 mutations [35], [37]. Interestingly, no basal increase in cell death and apoptosis was observed with PINK1 deficient differentiated human neurons or primary mouse neurons at early time points. However, molecular differences do exist between control and PINK1 kd cells at this early stage, specifically in their ability to handle mitochondrial stress. We find that PINK1 deficient cells are acutely sensitive to the toxin STS, which triggers mitochondrial apoptosis via opening of the mitochondrial permeability transition pore (MPTP) and subsequent cytochrome c release. In SHSY5Y cells lacking PINK1, Bax translocation to the mitochondria and cytochrome c release to the cytoplasm occurs earlier than in controls, and results in elevated levels of caspase activation (caspase-3 and caspase-9). In turn, increased levels of PARP cleavage and nuclear fragmentation are observed in PINK1 kd cells. Our findings are in line with previous reports demonstrating that PINK1 over-expression protects cells from STS-induced apoptosis [35] and transient RNAi-mediated PINK1 kd sensitises cells to various stressors [37], [38], [42], [43]. Our findings confirm the requirement of PINK1 within human neurons for the regulation of mitochondrial permeability transition, and prevention of caspase-3 mediated neuronal apoptosis. Our model shows some similarities to toxin based models of PD: the administration of MPTP to mice results in dopaminergic neuronal loss mediated by Bax translocation to the mitochondria, cytochrome c release, and activation of caspase 9 and caspase 3. In corroboration, over-expression of PINK1 in the SN of rats protects against the effects of MPTP [38]. In the MPTP model, the sensitivity to Bax-mediated apoptosis is determined by high levels of ROS (resulting from MPTP induced complex 1 deficiency) increasing the soluble pool of cytochrome c in the intermembrane space that may be released on stimulation by Bax. [44], [45] In our model, PINK1 deficiency is also associated with high levels of mitochondrial ROS production and thus the increased sensitivity to mitochondrial apoptosis may operate by a similar mechanism.

### PINK1 deficiency causes loss of mitochondrial membrane potential (ψm)

Mitochondrial dysfunction is strongly implicated in the pathogenesis of PD, either as a cause or downstream hallmark of dopaminergic degeneration [46]. PINK1 is targeted to mitochondria where it is has been proposed to maintain mitochondrial membrane potential (ψm), the driving force behind oxidative phosphorylation and ion transportation. Over-expression of wild type PINK1 in cell lines was found to protect cells from membrane depolarisation in response to proteasomal stress [27], [47]. Using live imaging techniques we show that PINK1 deficiency causes a reduction in the basal ψm in human neurons, implicating a role for PINK1 in the maintenance of membrane potential, even in the absence of stress. This is a significant finding as mitochondrial potential is critical for the synthesis of ATP through the dissipation of the transmembrane proton gradient. ATP availability is critical for cellular anabolism and importantly for neurons, maintaining a resting plasma membrane potential for cell excitability. A reduced proton gradient would explain decreased ATP levels in PINK1 fly knockout models [32], [34] and it would be interesting to investigate whether this is due to a reduced ψm *in vivo*.

### PINK1, ROS generation and glutathione levels

One important sequelae of mitochondrial dysfunction is increased generation of reactive oxygen species, which in turn can cause oxidative stress and damage to macromolecules within cells [11]. This damage extends to the mitochondrial respiratory components themselves, compromising function further. Evidence of oxidative stress and damage to proteins, lipids and nucleic acids has been found in the substantia nigra of sporadic PD patients and more recently, in fibroblasts from patients carrying PINK1 mutations [48]. We show here that in the absence of PINK1, ROS generation in both mitochondria and cytoplasm is increased, even in young human neurons. PINK1 knockout flies demonstrate increased sensitivity to oxidative stress [31], [33] and increased ROS generation and oxidative stress is similarly found in animal models of parkin and DJ-1 loss-of-function [49]–[52]. Hence ROS generation and elevated oxidative stress appears to be a unifying cellular phenotype in recessive Parkinsonism. This is the first demonstration that increased ROS production occurs early in a mammalian model of PD prior to neuronal stress and apoptosis, and hence suggests that it has an important role in the pathogenesis of neuronal dysfunction and death.

PINK1 may exert its protective effect on ψm and the prevention of ROS generation via phosphorylation of Tumor-necrosis factor associated protein-1 (TRAP1). TRAP1 (Hsp75) is an ATP-binding molecular chaperone co-localises with PINK1 in mitochondria, and is phosphorylated by PINK1 *in vitro* [43]. Silencing of TRAP1 in tumor cells also causes mitochondrial swelling, loss of ψm, rapid increase in intracellular ROS generation and release of cytochrome C, identical to the effects of PINK1 knockdown [43], [53]. Rescue experiments placed TRAP1 downstream of PINK1, with phosphorylation of TRAP1 required for its protective activity, which indirectly prevents the release of cytochrome C and ROS generation in response to apoptogenic agents such as hydrogen peroxide. The role of a putative PINK1-TRAP1 interaction in dopaminergic neurons has yet to be investigated, but could provide a key to selective degeneration of these cells if heavily utilised as a protective pathway during stress.

The primary mechanism for detoxification of ROS in the cytoplasm is the oxidation of reduced glutathione (GSH), which is regenerated harmlessly to the reduced species by the flavoenzyme glutathione reductase. We show that there is a significant decrease in steady state levels of total glutathione in primary human neurons lacking PINK1, which is likely to greatly impair their antioxidant defences. Evidence for decreased total GSH levels in the brains of patients with idiopathic PD has been widely reported and may occur as a result of decreased GSH synthesis, which is ATP dependent and thus compromised as a result of failing membrane potential [54]. Chronic inhibition of glutathione synthesis in dopaminergic neurons *in vitro* using buthionone-S-sulfoximime (BSO) reduced long term neuronal viability [55]. Numerous studies in rodents have demonstrated that glutathione deficiency by BSO administration results in mitochondrial swelling with vacuolization and rupture of the cristae and mitochondrial membranes seen by EM [56]. This may explain our observations of altered mitochondrial morphology in PINK1 deficient human neurons (see below). The importance of glutathione in recessive PD has already been explored: depleting parkin null flies of glutathione-S-transferase enzyme exacerbated the neurodegenerative phenotype [57]. Conversely, re-introduction of GST was found to rescue the mitochondrial swelling and neuronal loss in parkin knockouts [58]. Clearly, there exists a complex interrelationship between glutathione levels, respiratory chain impairment, mitochondrial morphology, and cell viability within neurons which both PINK1 and parkin co-operate [32].

### Altered mitochondrial integrity and proliferation

Interestingly a disrupted mitochondrial morphology was seen in aged human neurons lacking PINK1. Neuronal mitochondria appeared enlarged, with disrupted cristae and greatly reduced matrix volume. This finding corroborates the striking muscle phenotype in PINK1 and parkin knockout fly models, where myofibrils contain vacuolated, swollen and dysmorphic mitochondria, with disorganised christae [31], [32], [34]. Enlarged mitochondria were also seen in surviving dopaminergic neurons, the frequency of which increased with age [32]. Using genetic complementation experiments PINK1 was subsequently shown to function ‘up-stream’ of parkin to regulate mitochondrial integrity [32]. Recently, it was found that loss-of-function of the mitochondrial fission-promoting component Drp1, caused lethality in PINK1 or parkin knockout flies [59]. Conversely, over-expression of Drp1 or mutations in mitochondrial fusion-promoting proteins (Mitofusin2 and OPA1) suppressed the PINK1 and parkin null phenotype. These experiments suggest that these PD genes co-operate to regulate mitochondrial dynamics and that mitochondrial fission (splitting) is stimulated in response to inactivation of the PINK1-parkin pathway.

We provide interesting evidence for mitochondrial proliferation in PINK1 knockdown neurons, including increases in mean mitochondrial number per neuron, increased Mitotracker uptake, enhanced expression of respiratory chain complex subunits and elevated citrate synthase activity. Despite an increase in mass, no significant change in respiratory chain activity was detected in neurons lacking PINK1, implying that oxidative phosphorylation may be impaired in the increased numbers of mitochondria in these cells. It is established that increased levels of intracellular ROS may mediate changes in mitochondrial abundance and mtDNA copy number which occur during the ageing process [60]. The oxidative stress induced proliferation of mitochondria may initially be beneficial to compensate for dysfunctional mitochondria, and to supply ATP needed for cell survival. However increased numbers of mitochondria will also result in a further excess of ROS, which in the presence of insufficient antioxidant defense mechanisms, will produce more oxidative damage to cells. Increased oxidative damage will render neurons susceptible to death by apoptosis from mitochondrial membrane permeabilisation and cytochrome c release, or by necrosis due to insufficient ATP production. An increase in mitochondrial proliferation has so far not been reported in any other PD model.

### PINK1 deficiency and lysosomes

We also report here a novel phenotype in the surviving neurons of PINK1 deficient neurons which resembles the pathology observed in lysosomal storage diseases. Large intracellular bodies comprised of multivesicular ‘aggregates’ were detected in ageing PINK1 kd neurons, which were lysosomal in nature. PINK1 has not previously been implicated in lysosomal dysfunction but endogenous PINK1 expression has been detected in microsomes [35]. Lysosomes are present within autophagosomes, the multilamellar bodies responsible for proteolytic degradation of macromolecules and thought to be crucial in the clearance of amyloidogenic proteins such as α-synuclein and huntingtin [61]. Autophagy involves the non-selective degradation of proteins and organelles by lysosomes. However selective autophagy for certain damaged organelles such as mitochondria may occur in response to ROS. Moreover it has been proposed that mitochondria may act as a major source of the ROS signal required to activate autophagy. In our model of PINK1 deficiency we see an increase in autophagolysosomes suggesting an upregulation of autophagy. This may be occurring as a response to remove oxidatively damaged proteins and/or structurally abnormal mitochondria. The concurrent increase in cytosolic and mitochondrial ROS production may act as a signal for this process [62]. The lysosomal pathology we find in neurons resembles that seen in SHSY5Y cells over-expressing mutant β-synuclein protein [63]. β-synuclein is thought to be neuroprotective by interacting with and preventing the formation of α-synuclein inclusions. Pharmacological prevention of autophagy exacerbated cell death and α-synuclein toxicity, suggesting that they may fulfil a protective function in neurons. Enhancing formation of lysosomal clearance mechanisms in PD may therefore be a viable therapeutic strategy.

In summary, to study the role of PINK1 in PD, we have generated models of PINK1 loss of function in human and mouse neurons. PINK1 is up-regulated during neuronal differentiation and plays a major role in the neuroprotection of mature neurons. Neurons that lack PINK1 function are prone to apoptosis via the intrinsic mitochondrial apoptosis pathway. Moreover loss of PINK1 function results in increased levels of oxidative stress and reduced mitochondrial membrane potential, suggesting that mitochondrial dysfunction directly leads to the increased susceptibility to apoptosis. Further characterisation of the molecular events within mitochondria that lack PINK1 will enable a better understanding of the pathophysiologic mechanisms that cause the phenotype of PINK1 loss-of-function. Persistent mitochondrial dysfunction over time ultimately results in structural changes of the mitochondria which are reminiscent of PINK1 knockout fly models, but to date have not been detected in mammalian models.

To our knowledge this is the first study to provide a functional role for PINK1 in human neurons, and specifically midbrain derived neurons. Modelling human neurodegenerative disease may only be successfully achieved through recapitulation of the ageing process. Under these circumstances we find an age-specific phenotype for loss of PINK1 function. This provides unique insights into the pathogenesis of sporadic PD in which the phenotype of oxidative stress, mitochondrial dysfunction and neuronal apoptosis has been well established. Further it suggests a convergence of the molecular pathways of PINK1-associated PD and sporadic PD.

## Supporting Information

Methods S1 Supplementary Methods.
(0.04 MB DOC)

Table S1 (0.05 MB DOC)

Figure S1 A) Graph showing the increase in mRNA of different markers of dopamine neurons following differentiation of NSCs to neurons, using the pre-D method. Expression of TH, DAT, lmx1a, and Nurr1 are significantly elevated upon differentiation. Levels are normalized to housekeeping gene ATPB5, and shown relative to fetal brain tissue expression. B) Graph showing the effects of four different shRNA constructs on PINK1 gene expression in SHSY5Y cells. Values are mean RQs of five clones±s.e.m. Construct 1; sequence 1029, Construct 2; sequence 2194. Construct 3; 780, Construct 4; lamin AC. C) Graph showing knockdown effect of shRNA construct 1 (1029) on PINK1 gene expression in individual NSC clones normalized to expression levels with vector only. Values are mean RQ; plus and minus error bars represent maximum and minimum possible RQ value. D) Western blot showing endogenous PINK1 expression in primary cortical neurons in either wild type (WT), Heterozygote (Ht) or PINK1 knockout (KO) mouse primary cortical neurons. Anti-PINK1 (Novus) was used to detect PINK expression; β-actin levels are shown as loading control.
(0.89 MB TIF)

Figure S2 A) Histogram showing no significant increase in basal levels of apoptosis in PINK1 kd human NSCs compared to control cells after 24 h in culture. Levels of AnnexinV/PI staining were quantified using FACS. Values represent means of 3 independent experiments measured in triplicate±sem. B) Histogram demonstrating significant differences in live cell number, early apoptosis (annexin V only) and total apoptosis (annexin V+PI) in PINK1 kd SHSY5Y cells compared to controls. Cells were cultured for 96 hours before assaying using annexin V based FACS. Values represent mean values of 3 clones plated in duplicate and 20,000 cells measured per well±sem C) Double immunofluorescnece of primary embryonic mouse neuronal cultures using MAP2 (green), GFAP (red) and Hoechst (blue). Scale bar  =  20 µM. D) Comparison of individual cell parameters assayed by the Cytotoxicity algorithm for aged PINK1 KO mouse cortical neurons (day 30) compared to wild type controls, including mean nuclear size, mean nuclear intensity and lysosomal mass/pH. Values shown are means±s.e.m of 2 wild type and 3 KO cultures, each measured in triplicate.
(4.00 MB TIF)
