# Correction: PINK1 Is Necessary for Long Term Survival and Mitochondrial Function in Human Dopaminergic Neurons

**DOI:** 10.1371/annotation/17d5aaa1-c6d8-4aad-a9a4-56b2c1220c83

**Published:** 2008-07-22

**Authors:** Alison Wood-Kaczmar, Sonia Gandhi, Zhi Yao, Andrey Y. Abramov, Erik A. Miljan, Gregory Keen, Lee Stanyer, Iain Hargreaves, Kristina Klupsch, Emma Deas, Julian Downward, Louise Mansfield, Parmjit Jat, Joanne Taylor, Simon Heales, Michael R. Duchen, David Latchman, Sarah J. Tabrizi, Nicholas W. Wood

The fourth author's name appears incorrectly in the author byline and in the citation. It should read: Andrey Y. Abramov, and the citation should read: Wood-Kaczmar A, Gandhi S, Yao Z, Abramov AY, Miljan EA, et al. (2008) PINK1 Is Necessary for Long Term Survival and Mitochondrial Function in Human Dopaminergic Neurons. PLoS ONE 3(6): e2455. doi:10.1371/journal.pone.0002455

