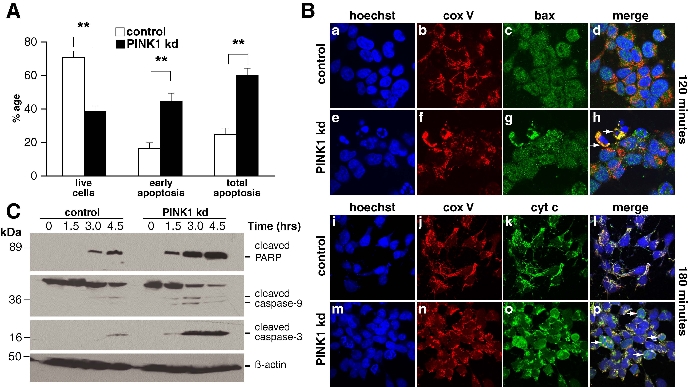# Correction: PINK1 Is Necessary for Long Term Survival and Mitochondrial Function in Human Dopaminergic Neurons

**DOI:** 10.1371/annotation/ba489c2a-5cf2-481c-aff7-d2c8c4ecdcfa

**Published:** 2008-08-21

**Authors:** Alison Wood-Kaczmar, Sonia Gandhi, Zhi Yao, Andrey Y. Abramov, Erik A. Miljan, Gregory Keen, Lee Stanyer, Iain Hargreaves, Kristina Klupsch, Emma Deas, Julian Downward, Louise Mansfield, Parmjit Jat, Joanne Taylor, Simon Heales, Michael R. Duchen, David Latchman, Sarah J. Tabrizi, Nicholas W. Wood

Figure 3Ba and 3Be are identical by mistake. Please view the entire corrected Figure 3 here:

**Figure:**