# Supplementary material for: PINK1 Is Necessary for Long Term Survival and Mitochondrial Function in Human Dopaminergic Neurons
Source: PLoS One. 2008 Jun 18;3(6):e2455. doi: 10.1371/journal.pone.0002455 (PMC2413012; doi:10.1371/journal.pone.0002455)
Supplement: Methods S1 — Supplementary Methods. (0.04 MB DOC) [file pone.0002455.s001.doc]

### Supplementary Methods

#### Generation of PINK1 knockout mice

The PINK1 locus was targeted by homologous recombination in mouse embryonic day 14.5 (E14.5) embryonic stem (ES) cells that were derived from the 129SvEvBrd strain (Lex-2) using standard ES cell culture and gene targeting techniques (Hogan et al. 1994; Joyner 1999). The targeting vector was pKO Scrambler NTKV-1901 (Stratagene), and the 5’ and 3’ arms of homology were generated by PCR. The 5’ arm of homology spans part of intron 1 to exon 2, and the 3’ arm of homology spans part of intron 5 to exon 8 of the PINK1 gene. Targeting the PINK1 locus with this vector inserts the IRES-lacZ/MC1neo in the second coding PINK1 exon at position bp 487 downstream of the translational start codon. The IRES-lacZ/MC1neo replaces most of exon 2, and all of exons 3-5 as well as intervening intronic sequences.

Chimeric mice resulting from the ES cell injections were bred to C57BL/6J albino mice in order to obtain germline transmission of the mutant PINK1 allele. Heterozygous PINK1 offspring (F1 generation) were interbred to generate F2’s, and F2 heterozygous mice were further backcrossed to C57Bl/6J / 129SvEv^Brd^ hybrid mice. These mice were then interbred to generate study populations of animals containing all three possible genotypes (wild type (WT), heterozygous (Het) and homozygous (KO) mutant). A PCR-based strategy was developed to distinguish WT and mutant PINK1 alleles. The following primers were used:

(PINK1-26): 5′-CTGCCCTCAGGGTCTCTAATGC

(PINK1-27): 5′-GGAAGGAGGCCATGGAAATTGT

(Neo): 5′-GCAGCGCATCGCCTTCTATC

A 193 bp product arising from primers Neo and PINK1-26 indicates the presence of the mutated allele, whereas a 296 bp product from primers PINK1-27 and PINK1-26 is amplified from the WT allele. Genomic DNA was isolated from tail or ear biopsy using the Extract-N-Amp Tissue PCR Kit (Sigma) and PCR conditions were: 94°C denaturation, 60°C annealing and 72°C extension for 45 sec each.

#### Primary embryonic mouse cortical cultures

Heads were collected in chilled dissection medium [HBSS without calcium or magnesium, supplemented with 0.45% (v/v) D-(+) glucose, 1 mM sodium pyruvate and 10 mM HEPES pH 7.4]. The whole brain was removed from the skull case in a Petri dish containing chilled dissection medium under sterile conditions. One cerebral hemisphere was dissected from each brain. The meninges of were removed and the cortices carefully excised and transferred to a sterile micro tube containing ~0.5 ml chilled dissection medium and allowed to settle under gravity. All dissection medium was removed from the cortices and replaced with 500 μl pre-warmed trypsin solution. The cortices were trypsinised for 15 minutes at 37˚C with gentle agitation halfway through incubation. The trypsination solution was then carefully aspirated and the cortices washed three times with 500 μl pre-warmed attachment medium [1x Modified Eagles Medium (MEM) with Earles and glutamine (Invitrogen), 1 mM pyruvic acid, 0.45% (w/v) D -(+) glucose (Sigma) and 10% (v/v) heat inactivated fetal bovine serum (Invitrogen)]. The last wash was replaced with 800 μl warm attachment medium and the tissue triturated using sterile fire-polished glass pipettes. Initially a pipette with pore diameter equivalent to a Gilson P1000 tip was used, followed by a pipette with pore diameter equivalent to a Gilson P200 tip, using a maximum of 15 passes with each or until a smooth cell suspension was produced. Cells were counted and plated at a density of 20,000 cells per well in pre-coated poly-L-lysine 96 well plates. Per embryo, 6-8 replicate wells were plated. Cells were then allowed to attach to the substrate for 3-4 h in a humidified CO_2_ incubator (5% CO_2_ in air) at 37°C. The attachment medium was then completely removed and replaced with prewarmed maintenance medium [Neurobasal medium (Invitrogen), 2% (v/v) B27 supplement, 2 mM glutamine, 100 I.U./ml penicillin and 100 I.U./ml streptomycin (Sigma) and 0.45% (v/v) D-(+) glucose]. Once the cells were in maintenance medium, half of the medium was replaced weekly. The remaining brain tissue from each dissected embryo was also carefully dissected and snap frozen for biochemical analysis.

#### SDS-PAGE and immunoblot analysis

Cells were lysed by the direct addition of preheated SDS sample buffer [0.125 M Tris-base pH 6.6, 2 % SDS (w/v), 10% sucrose (w/v), 2% (v/v) β-mercaptoethanol, 0.025% (w/v) Bromophenol blue]. Samples were separated on 10% or 16% Tris-Glycine mini gels using an XCell SureLock Electrophoresis system before being transferred onto Hybond nitrocellulose membranes (Amersham Biosciences) using an XCell Blot Module Version G. Membranes were incubated 5% (w/v) non-fat milk in PBS with 0.1% Tween-20(PBST) for 1 hour at RT/overnight at 4°C. Membranes were incubated in primary antibodies diluted in milk/PBST for 2 h at RT/overnight at 4°C (Table S1). Membranes were washed in PBST before incubating with appropriate HRP-conjugated secondary antibodies (Dako) diluted 1:1000-3000 in PBST. Membranes were washed in PBST prior to detection of bands using SuperSignal West Pico Chemiluminescent Substrate (Pierce). Membranes were exposed to x-ray films (BioMax) which were developed using a Compact X4 by XOgraph (Imaging Systems). Films were and scanned and stored as TIFF files. Images were cropped and annotated using Adobe Photoshop. Equivalent protein laoding was confirmed by stripping membranes and re-probing with anti-β-actin antibody.

#### Production of stable cell lines

Early passage (P11) NSCs were seeded in 6 well plates (75- 100k per well) and grown to 50% confluency prior to transduction with viral supernatant (SN). Wells were infected in duplicate with SN containing PSR vector only, PSR containing PINK1 shRNA and PSR containing shRNA directed against another gene, Lamin AC (provided by Dr. L. Mansfield). Each well was incubated with 1.5ml viral SN, 0.5ml B27 medium with 8ug/ml polybrene (Sigma) in a 37°C incubator. ‘Mock’ wells were incubated with medium and polybrene only. After 6-8h, viral SN was replaced with fresh medium overnight for recovery. Viral infection was repeated 24 h later and incubated overnight. Each well was then trypsinised and split to 2-3 10 cm dishes. Puromycin selection (1 ug/ml) commenced when cells became 90% confluent. Initially media was changed every 24 h until 5-6 days, when all cells on ‘mock’ infection plates had died. Media containing fresh growth factors and Puromycin was subsequently changed every 2-3 days. Once visible with the naked eye and adequately separated, cell colonies were picked and transferred to 96 well plates. Once confluent, each clone from a 96 well was trypsinised and transferred to the well of a 6 well plate. When confluent, cells were again trypsinised; 90% was frozen down and 10% used to seed a fresh 6 well. Once confluent, cells were harvested for RNA extraction and RT-PCR.

**Measurement of dopaminergic markers in differentiated human neurons**

Total cell RNA was extracted using Cells to cDNA kit (Ambion). Real time quantitative PCR (qRT-PCR) amplifications were carried out in a 10µl reaction volume mixture containing LightCycler Taqman master mix (Roche), hydrolysis Universal Probe Library (UPL, Roche), 0.4µM primers and cDNA equivalent to 125ngs of reverse transcribed RNA. Reactions and analysis were performed using a LightCycler 480 (Roche). Data was normalized to the housekeeping gene ATP5B, and expressed relative to fetal brain cDNA (Stratagene).

#### Measurement of total and reduced glutathione

For GSH analysis, cell pellets and midbrain tissue were suspended in homogenisation buffer comprising of 320mM sucrose, 1mM K^+^ EDTA and 10mM Tris-HCl, pH 7.4 , and homogenised on ice. Homogenates were then freeze thawed three times in liquid nitrogen and disrupted by sonication on ice for 1 minute (5s on/off intervals) using a MSE Soniprep 150 set at an amplitude of 14μA. Homogenates were subsequently mixed (1:1) with 15 mM *ortho*-phosphoric acid and incubated on ice for 15 min. Following mixing and centrifugation (15,000 ×*g* for 10 min), 20 μl of supernatant was injected onto the HPLC system. Separation and electrochemical detection were based on the method described by Riederer *et al.* (1989). In order to ascertain the ratio of GSH to GSSG, GSSG was reduced to GSH by pretreatment of homogenates with 2 units of glutathione reductase and 80μM NADPH for 15 min at 37˚C prior to the addition of *ortho*-phosphoric acid. All glutathione measurements were taken within one month of freezing cells or brain homogenates and were related to protein content. The protein concentration of samples was determined using the Bio-Rad DC protein assay using bovine serum albumin (BSA) as standards.

Measurement of citrate synthase activities

Shepherd JA, Garland PB. The kinetic properties of citrate synthase from rat liver mitochondria. Biochem J. 1969; 114: 597-610

#### Live Cell Imaging

For imaging experiments, cells were bathed in a HEPES-buffered salt solution (HBSS) composed (mM): 156 NaCl, 3 KCl, 2MgSO_4_, 1.25 KH_2_PO_4_, 2 CaCl_2_, 10 glucose and 10 Hepes, pH adjusted to 7.35 with NaOH. MitoSOX (10 μM) was loaded for 10 min followed by washing. All dyes were loaded at room temperature. For measurement of rates of ROS generation with dihydroethidium (HEt, 10μM) the dye was present in all solutions throughout the experiments. No preincubation (‘loading’) was used in order to limit the intracellular accumulation of oxidized product. Confocal images were obtained using a Zeiss 510 uv-vis CLSM equipped with a META detection system and a 40x oil immersion objective. HEt or MitoSOX were excited using the 543 nm laser line and fluorescence measured using a 560nm longpass filter. MitoSOX localization to mitochondria is potential dependent and it undergoes redistribution in response to loss of mitochondrial potential. The signal measured immediately over mitochondria decreases with redistribution, that in the cytosol increases and the global signal may increase slightly due to the enhancement of fluorescence as ethidium binds to DNA. Therefore we have taken pains to measure MitoSOX signals only immediately over mitochondria where the signal will if anything underestimate the real signal if the mitochondria depolarize. Changes in signal intensity seen before, at or during the time of mitochondrial depolarization can be reasonably interpreted as signaling increased ROS generation in mitochondria. Signal changes after loss of potential cannot be distinguished from HEt signals, as the principle of operation is now exactly the same as HEt.

#### ETC activities

Activities of the ETC enzymes were carried out as described previously complex I [Ragan et al] complex II+III [King 1967]; complex IV (cytochrome c oxidase; EC 1.9.3.1) [Wharton et al, 1967]. All activities were expressed as a ratio to citrate synthase (CS) to correct for mitochondrial enrichment of the samples [Sheperd & Garland, 1967].

Ragan CI, Wilson MT, Darley-Usmar VM, et al. Subfractionation of mitochondria and isolation of the proteins of oxidative phosphorylation. In: Darley-Usmar VM, Rickwood D, Wilson MT. eds. Mitochondria, a practical approach. London: IRL Press, 1987: 79-112

King TS. Preparations of succinate-cytochrome c reductase and the cytochrome b-c_1_ particle, and reconstitution of succinate-cytochrome c reductase. Methods Enzymol. 1967; 10: 217-235

Wharton DC, Tzagoloff A. Cytochrome oxidase from beef heart mitochondria. Methods Enzymol. 1967; 10: 245-250

Shepherd JA, Garland PB. The kinetic properties of citrate synthase from rat liver mitochondria. Biochem J. 1969; 114: 597-610
