# Supplementary material for: PINK1 Is Necessary for Long Term Survival and Mitochondrial Function in Human Dopaminergic Neurons
Source: PLoS One. 2008 Jun 18;3(6):e2455. doi: 10.1371/journal.pone.0002455 (PMC2413012; doi:10.1371/journal.pone.0002455)
Supplement: Table S1 — (0.05 MB DOC) [file pone.0002455.s002.doc]

#### Table S1 Antibodies

| **Antibody** | **Host** | **Supplier** | **Dilution**  **Immunoblot** | **Dilution**  **IF** |
| --- | --- | --- | --- | --- |
| Anti-β-actin | M^a^ | Sigma | 1:5000 | - |
| Anti-Bax | Rb | Santa Cruz (N-20) | 1:500 | 1:150 |
| Anti-PINK1 505A | Rb^b^ | Novus | 1:500 | 1:50 |
| Anti-PINK1 49 | Rb | In house^c^ | - | 1:50 |
| Anti-cleaved caspase-3 | Rb | R&D | 1:200 | - |
| Anti-caspase-9 pAb | M | R&D | 1:200 | - |
| Anti cleaved PARP | Rb | BioVision | 1:200 | - |
| Anti-βIII tubulin mAb | M | Chemicon | - | 1:500 |
| Anti-Tyrosine hydroxylase | Rb | Chemicon | - | 1:250 |
| Anti-Glial Fibrillary Acidic protein | M | DakoCytomation | - | 1:100 |
| Anti-Neurofilament 200 | M | Sigma | - | 1:50 |
| Anti Total OXPHOS complexes detection kit | M (5) | MitoSciences | 1:2500 | - |

^a^ Mouse

^b^Rabbit

^c^ See reference [21]
