# Supplementary figures and images for: PINK1 Is Necessary for Long Term Survival and Mitochondrial Function in Human Dopaminergic Neurons
Source: PLoS One. 2008 Jun 18;3(6):e2455. doi: 10.1371/journal.pone.0002455 (PMC2413012; doi:10.1371/journal.pone.0002455)

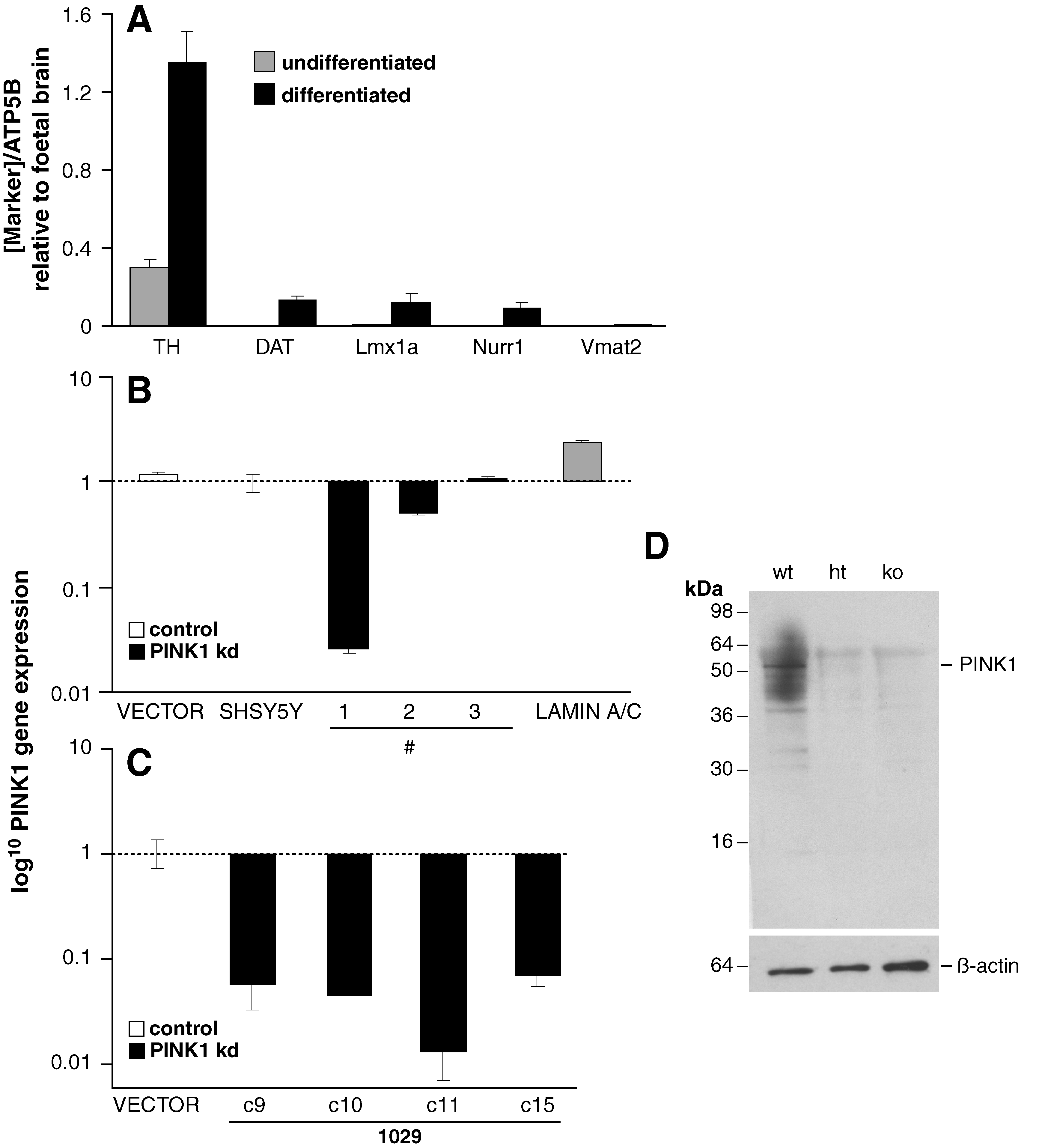

Supplement: Figure S1 — A) Graph showing the increase in mRNA of different markers of dopamine neurons following differentiation of NSCs to neurons, using the pre-D method. Expression of TH, DAT, lmx1a, and Nurr1 are significantly elevated upon differentiation. Levels are normalized to housekeeping gene ATPB5, and shown relative to fetal brain tissue expression. B) Graph showing the effects of four different shRNA constructs on PINK1 gene expression in SHSY5Y cells. Values are mean RQs of five clones±s.e.m. Construct 1; sequence 1029, Construct 2; sequence 2194. Construct 3; 780, Construct 4; lamin AC. C) Graph showing knockdown effect of shRNA construct 1 (1029) on PINK1 gene expression in individual NSC clones normalized to expression levels with vector only. Values are mean RQ; plus and minus error bars represent maximum and minimum possible RQ value. D) Western blot showing endogenous PINK1 expression in primary cortical neurons in either wild type (WT), Heterozygote (Ht) or PINK1 knockout (KO) mouse primary cortical neurons. Anti-PINK1 (Novus) was used to detect PINK expression; β-actin levels are shown as loading control. (0.89 MB TIF) [file pone.0002455.s003.tif]

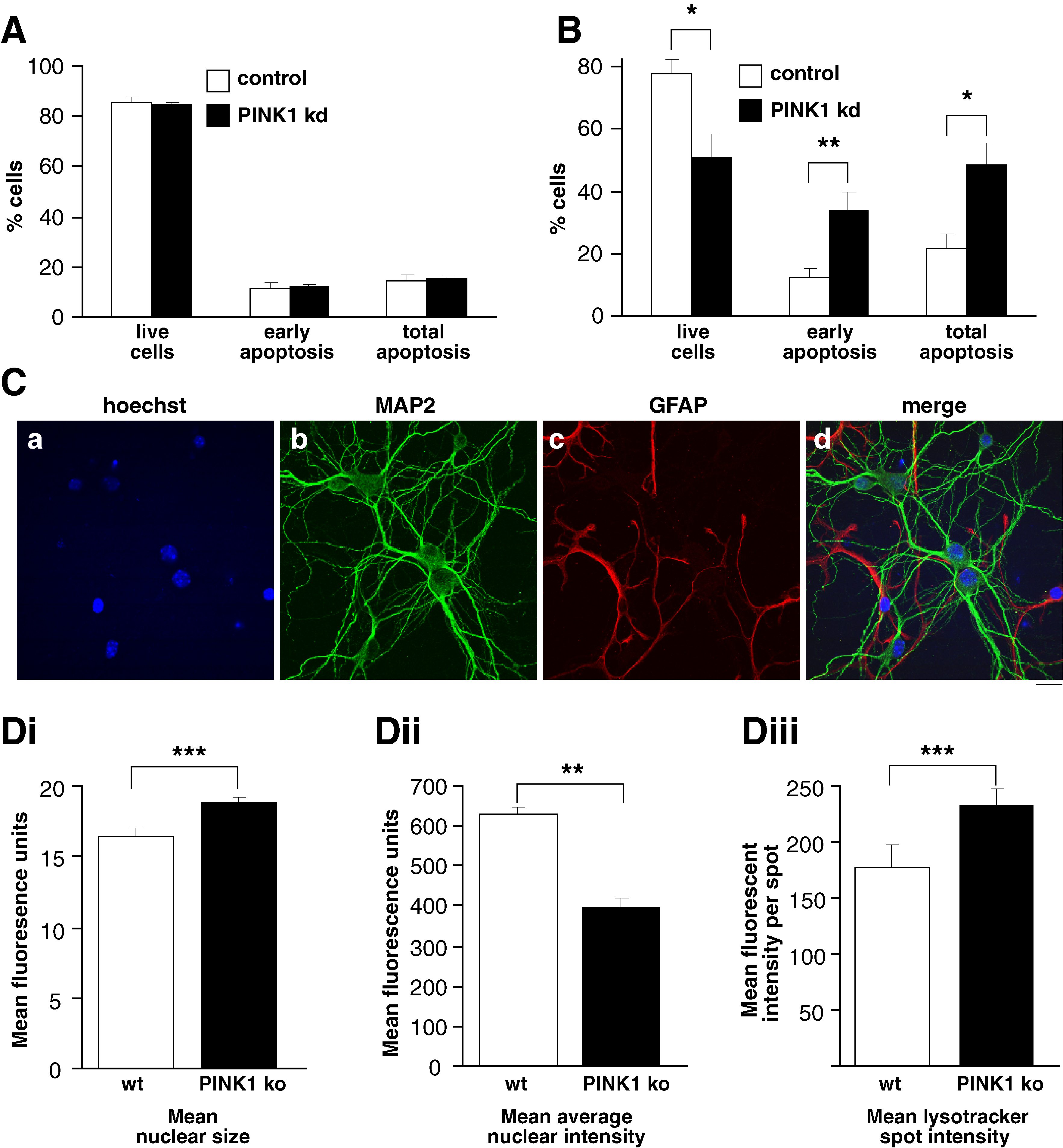

Supplement: Figure S2 — A) Histogram showing no significant increase in basal levels of apoptosis in PINK1 kd human NSCs compared to control cells after 24 h in culture. Levels of AnnexinV/PI staining were quantified using FACS. Values represent means of 3 independent experiments measured in triplicate±sem. B) Histogram demonstrating significant differences in live cell number, early apoptosis (annexin V only) and total apoptosis (annexin V+PI) in PINK1 kd SHSY5Y cells compared to controls. Cells were cultured for 96 hours before assaying using annexin V based FACS. Values represent mean values of 3 clones plated in duplicate and 20,000 cells measured per well±sem C) Double immunofluorescnece of primary embryonic mouse neuronal cultures using MAP2 (green), GFAP (red) and Hoechst (blue). Scale bar = 20 µM. D) Comparison of individual cell parameters assayed by the Cytotoxicity algorithm for aged PINK1 KO mouse cortical neurons (day 30) compared to wild type controls, including mean nuclear size, mean nuclear intensity and lysosomal mass/pH. Values shown are means±s.e.m of 2 wild type and 3 KO cultures, each measured in triplicate. (4.00 MB TIF) [file pone.0002455.s004.tif]
